# Prevalence of overweight and obesity, and associations with socio-demographic factors in Kuwait

**DOI:** 10.1186/s12889-021-10692-1

**Published:** 2021-04-07

**Authors:** Victor M. Oguoma, Neil T. Coffee, Saad Alsharrah, Mohamed Abu-Farha, Faisal H. Al-Refaei, Fahd Al-Mulla, Mark Daniel

**Affiliations:** 1 Australian Geospatial Health Laboratory, Health Research Institute, University of Canberra, Canberra, Australia; 2 Geohealth Laboratory, Dasman Diabetes Institute, Kuwait City, Kuwait; 3 Child Health Division, Menzies School of Health Research, Charles Darwin University, Darwin, Australia; 4 Biochemistry and Molecular Biology Department, Dasman Diabetes Institute, Kuwait City, Kuwait; 5 Department of Medicine, St. Vincent’s Hospital, The University of Melbourne, Melbourne, Australia

**Keywords:** Overweight, Obesity, Socio-demographic factors, Kuwait

## Abstract

**Background:**

Kuwait is amongst countries in the Gulf region with high income economy. According to the World Health Organisation (WHO), one in five adults in the Gulf region is obese. This study sought to evaluate the prevalence and magnitude of association between overweight, obesity, central obesity, and socio-demographic factors in Kuwait.

**Methods:**

A population-based cross-sectional survey of diabetes and obesity in Kuwait – part of the Kuwait Diabetes Epidemiology Program – was conducted between 2011 and 2014, targeting adults aged 18–82 years using the WHO STEPwise approach to non-communicable disease surveillance. Body mass index (BMI) was calculated to classify overweight and obesity, and waist circumference (WC) used to express central obesity. Multivariable logistic regression was used to estimate relationships between socio-demographic factors, overweight (25.0–29.9 kg/m^2^), obesity (≥30.0 kg/m^2^) or central obesity (WC ≥ 80 cm women; WC ≥ 94 cm men).

**Results:**

Records for gender (56% Men), age, BMI, governorate, and nationality existed for 4901 individuals. Mean age and BMI were 43 years and 30 kg/m^2^, respectively. Non-Kuwaiti nationals were more prevalent than Kuwaitis (76% vs 24%). Prevalence rates for overweight, obesity and central obesity were 40.6% (95%CI: 38.4–42.8%), 42.1% (95%CI: 40.0–44.3%) and 73.7% (95%CI: 71.7–75.6%), respectively. The youngest age group (18–29 years) had rates of 38.2% (95%CI: 29.2–47.7%), 27.2% (95%CI: 19.0–36.7%) and 49.9% (95%CI: 40.6–59.1%) for overweight, obesity and central obesity, respectively. In covariate-adjusted analyses, the odds of being overweight was 26% greater for men than for women. Conversely, women had a 54% (95%CI: 19–99%) and 7-fold (95%CI, 5–10-fold) greater odds of obesity/central obesity, respectively, than men. Greater educational attainment, physical activity, and non-Kuwaiti status were associated with lower odds of obesity/central obesity. History of smoking, elevated blood pressure, higher income, being married, greater age and female sex related to greater odds of obesity/central obesity.

**Conclusion:**

Overweight was greater in men, obesity greater in women. Overweight and obesity prevalence were high in young adults aged 18–29 years, a significant public health concern. Efforts to integrate mandatory physical education to the school curriculum and promoting the creation of recreation spaces/parks to promote physical activities, will play a vital role in the early prevention of overweight/obesity in Kuwait.

**Supplementary Information:**

The online version contains supplementary material available at 10.1186/s12889-021-10692-1.

## Background

Overweight and obesity (OW/OB) represent excess accumulations of adipose tissue associated with impaired physical as well as psychosocial health and well-being [1, 2]. Across different countries of the world, overweight and obesity are recognised as important public health problems. Decades of evidence show that obese individuals have higher risk of all-cause mortality; a constellation of serious health conditions and diseases such as type 2 diabetes, hypertension, dyslipidaemia, coronary heart disease, stroke, obstructive sleep apnoea, cancers and breathing complications, and difficulty with physical functioning and low quality of life [3–7]. More than 1.9 billion and 650 million adults worldwide aged 18 years and older are overweight and obese, respectively, and the number of deaths attributed to overweight and obesity is greater than that linked to underweight [1].

Kuwait is amongst countries in the Gulf Cooperation Council (GCC) with high income economy. According to the World Health Organisation (WHO), one in five adults in the GCC region is obese. The Global Health Observatory (GHO) data has documented that the age-standardised prevalence rates of overweight and obesity in Kuwait have increased from 51.4 and 18.6% in 1975 to 73.4 and 37.9% in 2016, respectively [8]. A recent cross-sectional study [9] of Kuwait reported for men and women respectively a median body mass index (BMI) of 28 kg/m^2^ and 29 kg/m^2^, and obesity rates of 36.5 and 44.0%.

A review of the noncommunicable disease profile of Kuwait shows that no operational policy, strategy, or action plan to reduce OW/OB and physical inactivity exits at this time [10, 11]. Further, given few studies addressing OW/OB in Kuwait, heterogeneity in how OW/OB have been reported for Kuwait, and an acknowledged international need for ethnic criteria for expressing excess adiposity for unique populations, there is a need to evaluate optimal anthropometric indices for tracking OW/OB in Kuwait [12].

Kuwait is an oil-rich country with significant population of expatriates, and has experienced rapid economic growth and major socio-demographic shifts concomitant with an epidemiological transition in extent of OW/OB over the last seven decades [13]. Non-Kuwaiti’s, largely an expatriate population, constitute about 67% of the population of Kuwait. In 2003, Al-Asi [14] reported that both OW/OB and physical inactivity are highly prevalent among expatriates in Kuwait. Kuwaitis, however, are the population segment benefitting most from rapid economic growth. An understanding of the magnitude of OW/OB between Kuwaiti and non-Kuwaiti residents is important to inform the development of interventions and obesity control resource allocation.

The WHO defines overweight and obesity as body mass index (BMI) of 25–29.9 kg/m^2^ and ≥ 30 kg/m^2^, respectively. Promoting a different measure, the International Diabetes Federation (IDF) emphasises excess central (or truncal) obesity using waist circumference (WC) for which the IDF suggests the criterion value, for the Middle East and Mediterranean region, be based on the European threshold, at least until specific data are available for this region [15]. This study sought to contribute data on obesity in the GCC region and to assess the extent of obesity between Kuwaiti and non-Kuwaiti ethnic residents using different anthropometric indices. We also sought to evaluate the prevalence and magnitude of association between overweight, obesity, central obesity, and socio-demographic factors in Kuwait.

## Methods

### Study design and participants

A population-based cross-sectional survey of diabetes and obesity in Kuwait – part of the Kuwait Diabetes Epidemiology Program – was conducted between 2011 to 2014, targeting adults aged 18–82 years. In 2011, the population of Kuwait above 18 years of age (based on the Public Authority of Civil Information (PACI) database) was 2,796,274; comprising of 670,432 Kuwaiti and 2,125,842 non-Kuwaiti’s living in the six governorates. A stratified random sampling technique was used to estimate diabetes prevalence for study participants. Using a computerised register developed by PACI, each of the six Kuwait governorates (Ahmadi, Capital, Farwaniya, Hawally, Jahra, and Mubarak Al Kabeer) was stratified into Kuwaiti and non-Kuwaiti majorities, yielding 12 strata. Within each stratum, a simple random sample of participants was selected to be proportional to the total numbers of the dominant population in each stratum. Stratum-specific sampling assumed a diabetes prevalence of 20% [16] with a 5% margin of error and 95% confidence level. Accounting for an anticipated 40% nonresponse rate, the total adjusted sample size sought was 4917.

### Demographics and questionnaire-based variables

The WHO STEPS questionnaire for non-communicable diseases surveillance was used to collect information on socio-demographic factors, behavioural characteristics (tobacco and alcohol use, diet, level of physical activity, and history of diseases), anthropometric measurements and biochemical parameters for participants [17]. Self-reported socio-demographic and individual characteristics, including gender, age, nationality, ethnicity, average income earnings reported in Kuwaiti Dinah, highest level of education completed, employment status, marital status, smoking history, and physical activity status, were recorded during a face-to-face interview at the Dasman Diabetes Institute. Physical activity questions were derived from the Global Physical Activity Questionnaire [18]. These asked whether participants’ work involves moderate-intensity activity and vigorous-intensity activity with small increases in breathing or heart rate such as brisk walking (or carrying light loads) for at least 10 min continuously.

### Anthropometric and physical measurements

Height and weight measured using a human digital column weighing scale with a mounted stadiometer (SECA, Germany), were used to define the BMI [19]. BMI was categorised as underweight (< 18.5 kg/m^2^), normal weight (18.5–24.9 kg/m^2^), overweight (25.0–29.9 kg/m^2^) and obese (> 30 kg/m^2^). The obese category was further subdivided into three classes: Obese class I (35.0–39.9 kg/m^2^), Obese class II (35.0–39.9 kg/m^2^) and Obese class III (≥40.0 kg/m^2^) [20].

Central obesity was assessed using waist circumference (WC). WC was measured using a constant tension tape, with arms relaxed at the sides, the highest point of the iliac crest and the mid-axillary line. The WC threshold for Europid ethnicity recommended for individuals from the Middle East and Mediterranean region by the Joint Scientific Statement on Harmonising the Metabolic Syndrome [21] was used. In men, WC ≥94 cm was classified as obesity, in women WC ≥80 cm. Additional anthropometric indices, waist-to-hip ratio and wait-to-height ratio, were calculated.

Blood pressure was measured using an Omron HEM-907XL digital sphygmomanometer (Omron Healthcare, Inc., Vernon Hills, IL, USA). Average of three readings of systolic and diastolic blood pressure was used to define an individual’s systolic and diastolic blood pressure readings. Elevated blood pressure was defined for both the American College of Cardiology/American Heart Association Hypertension (ACC/AHA) guideline (early high blood pressure = 130/80 mmHg] [22]) and the World Health Organisation (WHO) cut point (140/90 mmHg] [12]).

### Statistical analysis

Sampling weight was calculated by dividing the stratum-specific total sample population in the year 2011 by the estimated sample size for each stratum, based on the proportional allocation sampling approach. Statistical analysis was conducted using Stata 16.1 (StataCorp, College Station, TX, USA) using the survey estimation command. Descriptive statistics were calculated, stratified by age group and gender. Normally distributed continuous variables are presented as mean and standard deviation or standard error; categorical variables are presented as counts and percentages.

The Taylor-linearized variance estimation and Clopper-Pearson exact 95% confidence interval (CI) were used to derive prevalence estimates for overweight, obesity and central obesity. Straightforward categories of overweight (25–29.9 kg/m^2^) or obese (≥30 kg/m^2^) were created. Logistic regression was used to assess predictors of overweight, obesity and central obesity. Univariate logistic regression was conducted to identify socio-demographic factors and behaviours associated with overweight or obesity. Factors having a statistical significance of *p* < 0.25 were used in fitting a multivariable logistic regression solution using overweight, obesity, or central obesity as separate outcomes. Each of the predictors that constitute the fully adjusted model were included in a sequential order and model performance assessed using the Akaike Information criterion (AIC). Only the fully adjusted models were reported. No multiple imputation of missing data was performed; only the observed data were analysed. Statistical significance was set at *p* < 0.05. A choropleth map of the prevalence of overweight, obesity/central obesity was created using QGIS version 3.12.1 – a free and open-source software.

## Results

### Baseline characteristics of study population

The sample size surveyed was 5291. Table 1 presents sample characteristics stratified by gender. Four thousand nine hundred one (92.6%) individuals had valid records of which 57% were men, and 43% women. The weighted mean age of participants was 43 years. The population comprised predominantly middle-aged individuals with the highest proportion (37%) aged 40–49 years. The weighted percentages for the youngest (19–29 years) and oldest categories (> 60 years) were 9 and 6%, respectively. There were more non-Kuwaiti than Kuwaiti (weighted: 76% vs 24%) in the study population. Forty-eight percent (weighted) of the population had completed a bachelor’s degree, 51% are women. The average weighted weight and waist circumference were 82 kg and 98 cm, respectively, higher in men compared to women.

**Table 1:** Study characteristics by gender

Characteristics	Unweighted			Weighted		
	Male	Female	Total	Male	Female	Total
	***N*** = 2750	***N*** = 2151	***N*** = 4901	***N*** = 1,694,841	***N =*** 1,101,433	***N =*** 2,796,274
	n (%)	n (%)	n (%)	n (%)	n (%)	n (%)
**Age (years)** mean (SD or SE)	44.6 (10.4)	43.3 (10.3)	44.0 (10.4)	43.2 (0.3)	42.0 (0.3)	42.7 (0.2)
**Age group**						
18–29	191 (6.9)	181 (8.4)	372 (7.6)	152,751 (9.0)	101,688 (9.2)	254,440 (9.1)
30–39	688 (25.0)	618 (28.7)	1306 (26.6)	455,229 (26.9)	362,855 (32.9)	818,084 (29.3)
40–49	1010 (36.7)	798 (37.1)	1808 (36.9)	639,939 (37.8)	392,937 (35.7)	1,032,876 (36.9)
50–59	642 (23.3)	385 (17.9)	1027 (21.0)	345,495 (20.4)	182,359 (16.6)	527,854 (18.9)
≥ 60	219 (8.0)	169 (7.9)	388 (7.9)	101,426 (6.0)	61,594 (5.6)	163,020 (5.8)
**Nationality**						
Kuwaiti	867 (31.5)	822 (38.2)	1689 (34.5)	340,897 (20.1)	329,535 (29.9)	670,432 (24.0)
non-Kuwaiti, specify	1883 (68.5)	1329 (61.8)	3212 (65.5)	1,353,945 (79.9)	771,897 (70.1)	2,125,842 (76.0)
**Ethnicity**						
Arab	1741 (63.6)	1492 (69.5)	3233 (66.2)	932,282 (55.2)	689,155 (62.7)	1,621,436 (58.2)
Iranian	100 (3.7)	68 (3.2)	168 (3.4)	74,380 (4.4)	43,603 (4.0)	117,983 (4.2)
South Asian	744 (27.2)	303 (14.1)	1047 (21.4)	560,374 (33.2)	164,237 (14.9)	724,611 (26.0)
Southeast Asia	139 (5.1)	268 (12.5)	407 (8.3)	107,873 (6.4)	193,709 (17.6)	301,582 (10.8)
Other	15 (0.5)	15 (0.7)	30 (0.6)	13,497 (0.8)	8969 (0.8)	22,466 (0.8)
**Average Income**						
≤ 500	826 (35.5)	517 (31.4)	1343 (33.8)	668,554 (47.3)	302,892 (37.5)	971,446 (43.7)
> 500–1500	784 (33.7)	532 (32.3)	1316 (33.1)	442,577 (31.3)	266,998 (33.1)	709,575 (31.9)
> 1500	716 (30.8)	598 (36.3)	1314 (33.1)	303,009 (21.4)	237,961 (29.5)	540,970 (24.3)
**Education**						
Illiterate	17 (0.6)	40 (1.9)	57 (1.2)	8148 (0.5)	40,831 (3.7)	48,980 (1.8)
Read and write	469 (17.1)	360 (16.8)	829 (17.0)	323,163 (19.1)	206,017 (18.7)	529,181 (19.0)
Secondary School	711 (25.9)	487 (22.7)	1198 (24.5)	448,744 (26.6)	250,252 (22.7)	698,996 (25.1)
University	1305 (47.6)	1157 (53.9)	2462 (50.3)	774,114 (45.8)	556,085 (50.5)	1,330,199 (47.7)
High studies	240 (8.8)	104 (4.8)	344 (7.0)	135,937 (8.0)	47,008 (4.3)	182,945 (6.6)
**Occupation**						
Employed	2416 (88.5)	1219 (56.8)	3635 (74.5)	1,547,480 (92.1)	627,081 (57.0)	2,174,562 (78.2)
Student, not employed	16 (0.6)	24 (1.1)	40 (0.8)	9368 (0.6)	15,264 (1.4)	24,632 (0.9)
Housewife, not employed	0 (0.0)	619 (28.8)	619 (12.7)	0 (0.0)	359,074 (32.7)	359,074 (12.9)
Retired	282 (10.3)	211 (9.8)	493 (10.1)	102,231 (6.1)	72,732 (6.6)	174,963 (6.3)
Unemployed	16 (0.6)	73 (3.4)	89 (1.8)	20,777 (1.2)	25,168 (2.3)	45,944 (1.7)
**Marital status**						
Never married	208 (7.6)	180 (8.4)	388 (7.9)	153,251 (9.0)	104,773 (9.5)	258,024 (9.2)
currently married	2497 (90.8)	1788 (83.2)	4285 (87.4)	1,524,389 (89.9)	920,290 (83.6)	2,444,679 (87.4)
Divorced	35 (1.3)	104 (4.8)	139 (2.8)	13,535 (0.8)	46,328 (4.2)	59,864 (2.1)
Widowed	10 (0.4)	78 (3.6)	88 (1.8)	3666 (0.2)	29,782 (2.7)	33,448 (1.2)
**Current smoker**						
No	1813 (66.1)	1916 (89.3)	3729 (76.3)	1,171,971 (69.3)	1,003,578 (91.3)	2,175,549 (78.0)
Yes	930 (33.9)	230 (10.7)	1160 (23.7)	518,974 (30.7)	96,096 (8.7)	615,070 (22.0)
**Past smoker**						
No	1319 (71.5)	1844 (97.1)	3163 (84.4)	829,021 (70.0)	965,412 (97.0)	1,794,433 (82.3)
Yes	527 (28.5)	56 (2.9)	583 (15.6)	355,031 (30.0)	29,984 (3.0)	385,015 (17.7)
**Vigorous-intensity physical activity**						
No	2681 (97.8)	2114 (98.4)	4795 (98.1)	1,649,847 (97.6)	1,072,917 (97.5)	2,722,764 (97.6)
Yes	61 (2.2)	34 (1.6)	95 (1.9)	40,838 (2.4)	27,275 (2.5)	68,114 (2.4)
**Moderate-intensity physical activity**						
No	2505 (91.5)	1984 (92.4)	4489 (91.9)	1,505,793 (89.2)	1,017,283 (92.5)	2,523,076 (90.5)
Yes	233 (8.5)	163 (7.6)	396 (8.1)	181,541 (10.8)	82,650 (7.5)	264,191 (9.5)
**Elevated blood pressure (ACC/AHA)**						
No	1596 (58.2)	1637 (76.3)	3233 (66.2)	947,366 (56.0)	819,426 (74.5)	1,766,792 (63.3)
Yes	1145 (41.8)	509 (23.7)	1654 (33.8)	743,060 (44.0)	280,248 (25.5)	1,023,307 (36.7)
**Elevated blood pressure (WHO)**						
No	2348 (85.7)	1960 (91.3)	4308 (88.2)	1,421,989 (84.1)	997,897 (90.7)	2,419,886 (86.7)
Yes	393 (14.3)	186 (8.7)	579 (11.8)	268,437 (15.9)	101,776 (9.3)	370,213 (13.3)
**Height (m);** mean (SD or SE)	1.7 (0.1)	1.6 (0.1)	1.7 (0.1)	1.7 (0.0)	1.6 (0.0)	1.7 (0.0)
**Weight (kg);** mean (SD or SE)	86.5 (17.5)	76.8 (17.1)	82.3 (18.0)	85.8 (0.6)	75.9 (0.5)	81.9 (0.4)
**Waist Circumference (cm);** mean (SD or SE)	99.5 (13.2)	95.3 (13.5)	97.7 (13.5)	99.1 (0.4)	95.3 (0.4)	97.6 (0.3)
**Hip Circumference (cm);** mean (SD or SE)	105.5 (10.7)	108.5 (12.8)	106.9 (11.8)	104.9 (0.3)	107.6 (0.3)	105.9 (0.2)
**Waist to height ratio;** mean (SD or SE)	0.6 (0.1)	0.6 (0.1)	0.6 (0.1)	0.6 (0.0)	0.6 (0.0)	0.6 (0.0)

Table S1 indicates a higher weighted proportion 55% of participants aged 18–29 years old earning less than five hundred Kuwaiti Dinah compared to older adults in other age groupings. Across age groups, university education outweighed other levels of education.

Table S2 indicates that the proportion of non-Kuwaiti with average monthly household income ≤KD1500 was higher compared to Kuwaiti, while Kuwaiti were most represented (weighted: 77%) in the high-income group (>KD1500). Across nationalities, most participants had a bachelor’s degree. In both unweighted and weighted descriptive analysis, more Kuwaiti than non-Kuwaiti had completed a university education (63% versus 44%, respectively).

### Prevalence of overweight, obesity and central obesity

**Fig. 1:**
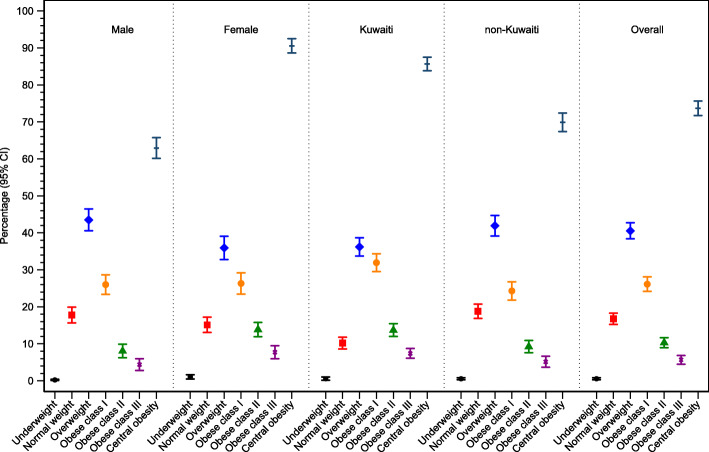
Prevalence of overweight and obesity stratified by gender, nationality, and overall

**Fig. 2:**
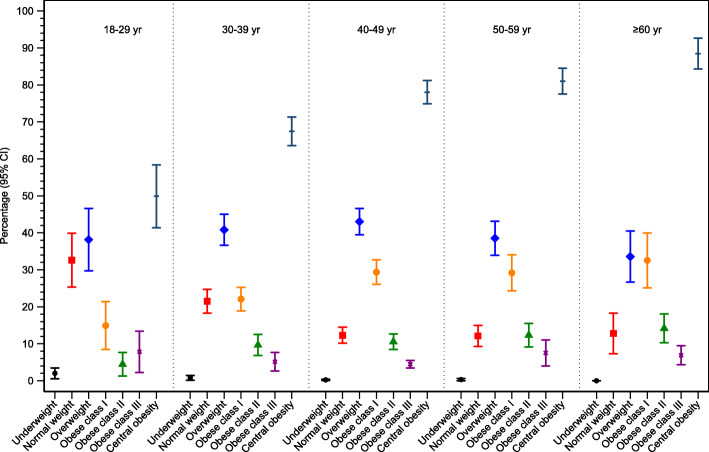
Prevalence of overweight and obesity stratified by age group

Figure 1 Weighted prevalence rates of overweight and obesity were, overall, 40.6% (95%CI: 38.4–42.8%) and 42.1% (95%CI: 40.0–44.3%), respectively. Obesity rates for obesity classes I, II and III were 26.1% (95%CI: 24.2–28.1%), 10.3% (95%CI: 9.0–11.6%), and 5.7% (95%CI: 4.5–6.9%), respectively. The prevalence of central obesity was 73.7% (95%CI: 71.7–75.6%). Men were more overweight 43.5% (95%: 40.6–46.4%) than women 35.9% (95%: 32.8–39.1%). Obesity and central obesity were greater in women (47.9 and 90.5%) relative to men (38.5 and 62.9%), respectively. Non-Kuwaiti’s were more overweight 41.9% (95%CI: 39.2–44.7%) than Kuwaiti’s 36.2% (95%CI: 33.7–38.7%). Obesity and central obesity were greater in Kuwaiti’s relative to non-Kuwaiti’s. Across age groups, overweight rose slightly from 19 to 29 years, plateaued between ages 30 to 59 and then declined at age > 60 years (Fig. 2). Obesity and central obesity both rose with increasing age.

**Fig. 3:**
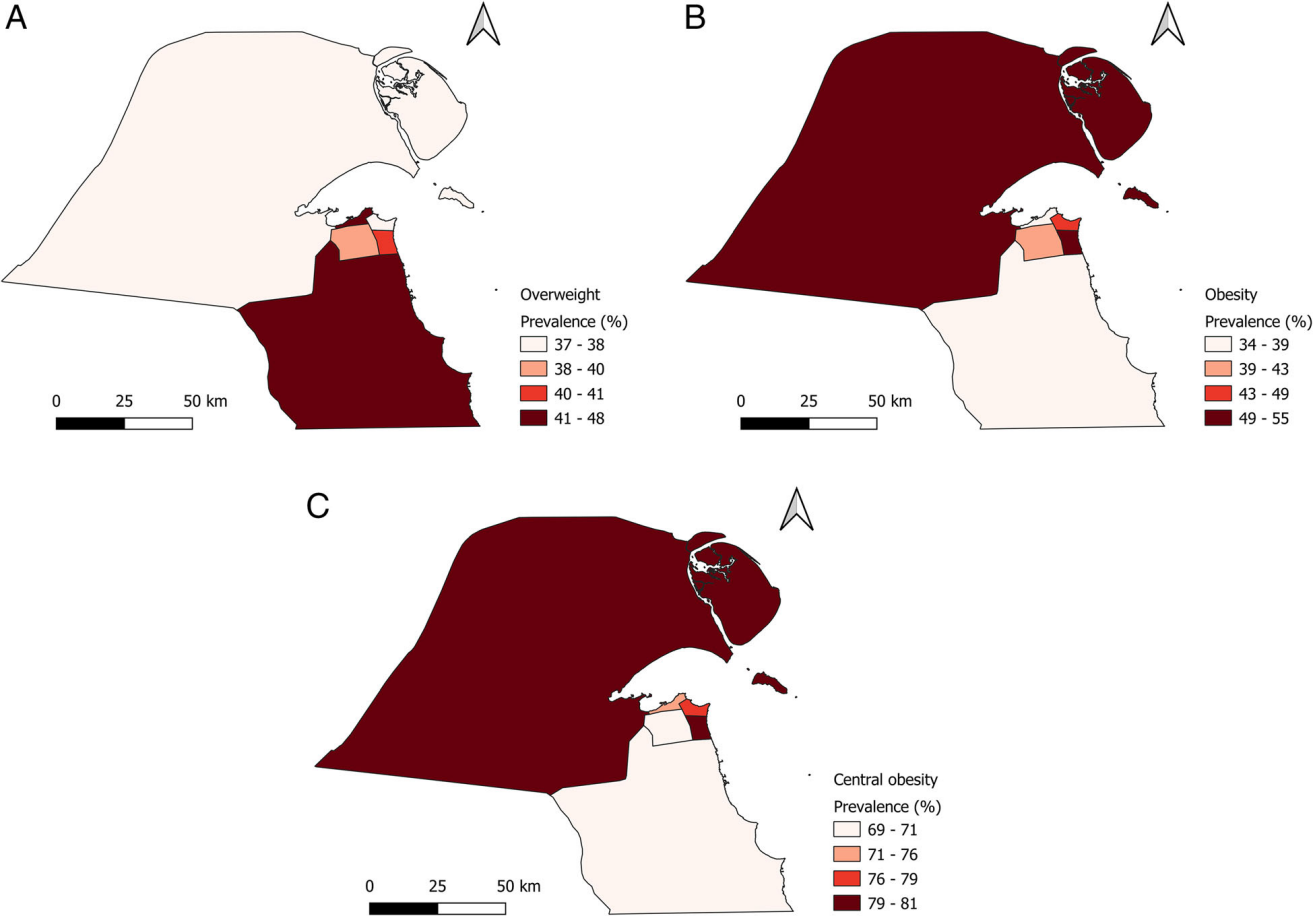
Prevalence of overweight and obesity by governorate. Map of the prevalence of overweight and obesity by governorate was created using free open-source QGIS software version 3.12.1

Figure 3 shows the choropleth map of the prevalence of overweight, obesity and central obesity according to governorate. Ahmadi 47.6% (95%CI: 41.7–52.3) had the highest prevalence of overweight, while Jahra had the highest prevalence of obesity and central obesity.

### Association of overweight, obesity and central obesity with socio-demographic factors

Table 2 provides univariate and multivariable analysis results for overweight. In adjusted analysis, women had lower odds of overweight [AOR = 0.79 (95%CI: 0.64–0.99); *p* = 0.04] compared to men. In univariate analysis, non-Kuwaiti had a 27% greater odds of overweight compared to Kuwaitis; this difference was attenuated, however, after accounting for sociodemographic factors. Individuals with average monthly household income > 1500 were less likely to be overweight compared to those who earn ≤500.

**Table 2:** Univariate and Multivariable analysis of association of overweight and study characteristics

	Univariate	*p*-value	Multivariable^a^	*p*-value
	OR (95% CI)		OR (95% CI)	
Gender				
Male	1.00 (1.00, 1.00)	.	1.00 (1.00, 1.00)	.
Female	0.72 (0.60, 0.86)	< 0.001	0.79 (0.64, 0.99)	0.04
**Age group**				
18–29	1.00 (1.00, 1.00)	.	1.00 (1.00, 1.00)	.
30–39	1.15 (0.77, 1.70)	0.50	1.16 (0.75, 1.78)	0.50
40–49	1.26 (0.86, 1.86)	0.23	1.37 (0.89, 2.11)	0.15
50–59	1.06 (0.71, 1.59)	0.77	1.23 (0.78, 1.96)	0.37
≥ 60	0.84 (0.53, 1.35)	0.48	0.88 (0.50, 1.54)	0.66
**Nationality**				
Kuwaiti	1.00 (1.00, 1.00)	.	1.00 (1.00, 1.00)	.
non-Kuwaiti, specify	1.27 (1.09, 1.48)	< 0.001	1.07 (0.80, 1.42)	0.65
**Education**				
Illiterate	1.00 (1.00, 1.00)	.	1.00 (1.00, 1.00)	.
Read and write	5.17 (1.79, 14.99)	< 0.001	2.11 (0.81, 5.54)	0.13
Secondary School	4.98 (1.73, 14.30)	< 0.001	1.81 (0.69, 4.74)	0.22
University	5.13 (1.81, 14.58)	< 0.001	2.14 (0.82, 5.55)	0.12
High studies	6.82 (2.31, 20.14)	< 0.001	2.43 (0.88, 6.68)	0.09
**Occupation**				
Employed	1.00 (1.00, 1.00)	.	1.00 (1.00, 1.00)	.
Student, not employed	1.26 (0.35, 4.47)	0.72	2.35 (0.49, 11.15)	0.28
Housewife, not employed	0.65 (0.48, 0.88)	0.01	0.75 (0.51, 1.10)	0.14
Retired	0.76 (0.61, 0.95)	0.02	1.03 (0.77, 1.38)	0.85
Unemployed	0.27 (0.13, 0.54)	< 0.001	0.82 (0.36, 1.87)	0.63
**Marital status**				
Never married	1.00 (1.00, 1.00)	.	–	–
currently married	1.17 (0.82, 1.67)	0.40	–	–
Divorced	0.95 (0.53, 1.70)	0.86	–	–
Widowed	0.94 (0.48, 1.85)	0.86	–	–
**Average Income**				
≤ 500	1.00 (1.00, 1.00)	.	1.00 (1.00, 1.00)	.
> 500–1500	0.87 (0.69, 1.11)	0.26	0.89 (0.68, 1.15)	0.37
> 1500	0.75 (0.61, 0.93)	0.01	0.80 (0.56, 1.13)	0.21
**Current smoker**				
No	1.00 (1.00, 1.00)	.	1.00 (1.00, 1.00)	.
Yes	1.15 (0.93, 1.42)	0.19	1.06 (0.84, 1.35)	0.60
**Past smoker**				
No	1.00 (1.00, 1.00)	.	–	–
Yes	0.91 (0.67, 1.24)	0.55	–	–
**Vigorous-intensity physical activity**				
No	1.00 (1.00, 1.00)	.	–	–
Yes	1.03 (0.56, 1.90)	0.93	–	–
**Moderate-intensity physical activity**				
No	1.00 (1.00, 1.00)	.	–	–
Yes	0.83 (0.57, 1.20)	0.32	–	–
**Elevated blood pressure (ACC/AHA)**				
No	1.00 (1.00, 1.00)	.	–	–
Yes	1.11 (0.91, 1.34)	0.30	–	–
**Elevated blood pressure (WHO)**				
No	1.00 (1.00, 1.00)	.	–	–
Yes	0.93 (0.70, 1.24)	0.62	–	–

^a^Adjusted for gender, age group, nationality, education, occupation, average monthly household income and current smoking status

Table 3 shows both univariate and adjusted analyses of obesity and central obesity. In the adjusted analysis, women had 54% (95%CI: 19–99%) and 7-fold (95%CI: 5.2 to 10.5-fold) greater odds of obesity and central obesity, respectively, compared to men, a substantial gender difference. The odds of obesity and central obesity also rose with advancing age. Respondents > 60 years had a 79% and 3.1-fold greater odds of obesity and central obesity, respectively, compared to the youngest age group (18–29 years) after accounting for socio-demographic factors. Non-Kuwaiti’s had a lower odd of obesity than Kuwaiti counterparts, but the difference was attenuated with adjustment for socio-demographic factors. Status as non-employed housewife was associated with a greater odd of being obese. Higher average income, previous history of smoking, and elevated blood pressure, were all associated with higher odds of obesity.

**Table 3:** Univariate and Multivariable analysis of association of obesity, Central Obesity, and study characteristics

Characteristics	Obesity				Central Obesity			
	Univariate		Multivariable^**a**^		Univariate		Multivariable^**b**^	
	OR (95% CI)	***p-***value	OR (95% CI)	***p-***value	OR (95% CI)	***p-***value	OR (95% CI)	***p-***value
Gender								
Male	1.00 (1.00, 1.00)	.	1.00 (1.00, 1.00)	.	1.00 (1.00, 1.00)	.	1.00 (1.00, 1.00)	.
Female	1.49 (1.24, 1.78)	< 0.001	1.54 (1.19, 1.99)	< 0.001	5.64 (4.37, 7.27)	< 0.001	7.39 (5.21, 10.49)	< 0.001
**Age group**								
18–29	1.00 (1.00, 1.00)	.	1.00 (1.00, 1.00)	.	1.00 (1.00, 1.00)	.	1.00 (1.00, 1.00)	.
30–39	1.42 (0.91, 2.21)	0.12	1.29 (0.75, 2.21)	0.36	2.08 (1.42, 3.06)	< 0.001	0.95 (0.48, 1.86)	0.88
40–49	1.93 (1.25, 2.97)	< 0.001	1.63 (0.95, 2.82)	0.08	3.57 (2.43, 5.25)	< 0.001	1.86 (0.92, 3.75)	0.08
50–59	2.30 (1.46, 3.64)	< 0.001	1.49 (0.83, 2.68)	0.18	4.29 (2.85, 6.46)	< 0.001	1.92 (0.91, 4.06)	0.09
≥ 60	2.76 (1.67, 4.56)	< 0.001	1.79 (0.90, 3.55)	0.10	7.71 (4.53, 13.15)	< 0.001	3.10 (1.24, 7.76)	0.02
**Nationality**								
Kuwaiti	1.00 (1.00, 1.00)	.	1.00 (1.00, 1.00)	.	1.00 (1.00, 1.00)	.	1.00 (1.00, 1.00)	.
non-Kuwaiti, specify	0.56 (0.48, 0.66)	< 0.001	0.75 (0.54, 1.05)	0.10	0.39 (0.32, 0.47)	< 0.001	0.58 (0.38, 0.87)	0.01
**Education**								
Illiterate	1.00 (1.00, 1.00)	.	1.00 (1.00, 1.00)	.	1.00 (1.00, 1.00)	.	1.00 (1.00, 1.00)	.
Read and write	0.17 (0.07, 0.42)	< 0.001	0.71 (0.27, 1.84)	0.48	0.15 (0.04, 0.53)	< 0.001	0.39 (0.06, 2.54)	0.33
Secondary School	0.15 (0.06, 0.38)	< 0.001	0.72 (0.28, 1.85)	0.50	0.14 (0.04, 0.50)	< 0.001	0.38 (0.06, 2.39)	0.30
University	0.14 (0.06, 0.34)	< 0.001	0.55 (0.22, 1.39)	0.21	0.13 (0.04, 0.45)	< 0.001	0.27 (0.04, 1.72)	0.16
High studies	0.11 (0.04, 0.29)	< 0.001	0.59 (0.22, 1.61)	0.30	0.09 (0.03, 0.33)	< 0.001	0.34 (0.05, 2.29)	0.27
**Occupation**								
Employed	1.00 (1.00, 1.00)	.	1.00 (1.00, 1.00)	.	1.00 (1.00, 1.00)	.	1.00 (1.00, 1.00)	.
Student, not employed	0.94 (0.29, 3.10)	0.92	0.30 (0.08, 1.13)	0.07	0.60 (0.15, 2.30)	0.45	0.15 (0.03, 0.71)	0.02
Housewife, not employed	2.42 (1.82, 3.23)	< 0.001	2.15 (1.42, 3.24)	< 0.001	12.19 (8.03, 18.51)	< 0.001	3.33 (1.51, 7.32)	< 0.001
Retired	2.08 (1.67, 2.58)	< 0.001	1.07 (0.77, 1.49)	0.70	3.80 (2.72, 5.30)	< 0.001	1.25 (0.72, 2.16)	0.44
Unemployed	3.86 (1.85, 8.05)	< 0.001	1.00 (0.38, 2.66)	1.00	3.62 (1.32, 9.92)	0.01	0.69 (0.21, 2.23)	0.54
**Marital status**								
Never married	1.00 (1.00, 1.00)	.	1.00 (1.00, 1.00)	.	1.00 (1.00, 1.00)	.	1.00 (1.00, 1.00)	.
currently married	2.79 (1.90, 4.10)	< 0.001	2.24 (1.35, 3.70)	< 0.001	4.11 (2.95, 5.72)	< 0.001	2.78 (1.68, 4.60)	< 0.001
Divorced	2.85 (1.64, 4.94)	< 0.001	1.82 (0.86, 3.86)	0.12	9.18 (4.93, 17.10)	< 0.001	2.59 (1.05, 6.42)	0.04
Widowed	4.02 (2.08, 7.77)	< 0.001	1.51 (0.56, 4.08)	0.42	17.92 (6.17, 52.01)	< 0.001	3.97 (0.59, 26.96)	0.16
**Average Income**								
≤ 500	1.00 (1.00, 1.00)	.	1.00 (1.00, 1.00)	.	1.00 (1.00, 1.00)	.	1.00 (1.00, 1.00)	.
> 500–1500	1.84 (1.44, 2.36)	< 0.001	1.78 (1.29, 2.45)	< 0.001	1.98 (1.51, 2.59)	< 0.001	1.75 (1.23, 2.50)	< 0.001
> 1500	2.46 (1.95, 3.09)	< 0.001	2.17 (1.41, 3.33)	< 0.001	3.32 (2.59, 4.25)	< 0.001	2.25 (1.40, 3.61)	< 0.001
**Current smoker**								
No	1.00 (1.00, 1.00)	.	1.00 (1.00, 1.00)	.	1.00 (1.00, 1.00)	.	1.00 (1.00, 1.00)	.
Yes	0.85 (0.69, 1.05)	0.13	2.54 (1.02, 6.36)	0.05	0.70 (0.56, 0.88)	< 0.001	4.61 (1.70, 12.49)	< 0.001
**Past smoker**								
No	1.00 (1.00, 1.00)	.	1.00 (1.00, 1.00)	.	1.00 (1.00, 1.00)	.	1.00 (1.00, 1.00)	.
Yes	1.21 (0.89, 1.64)	0.22	1.45 (1.02, 2.04)	0.04	0.80 (0.58, 1.12)	0.20	1.43 (0.96, 2.13)	0.07
**Vigorous-intensity physical activity**								
No	1.00 (1.00, 1.00)	.	–	–	1.00 (1.00, 1.00)	.	–	–
Yes	0.68 (0.32, 1.45)	0.32	–	–	0.76 (0.42, 1.36)	0.35	–	–
**Moderate-intensity physical activity**								
No	1.00 (1.00, 1.00)	.	–	–	1.00 (1.00, 1.00)	.	1.00 (1.00, 1.00)	.
Yes	0.99 (0.68, 1.43)	0.96	–	–	0.64 (0.43, 0.94)	0.02	0.79 (0.45, 1.38)	0.40
**Elevated blood pressure (ACC/AHA)**								
No	1.00 (1.00, 1.00)	.	–	–	1.00 (1.00, 1.00)	.	–	–
Yes	1.29 (1.07, 1.57)	0.01	–	–	1.16 (0.94, 1.44)	0.18	–	–
**Elevated blood pressure (WHO)**								
No	1.00 (1.00, 1.00)	.	1.00 (1.00, 1.00)	.	1.00 (1.00, 1.00)	.	1.00 (1.00, 1.00)	.
Yes	1.49 (1.12, 1.97)	0.01	2.34 (1.59, 3.44)	< 0.001	1.26 (0.91, 1.76)	0.17	2.13 (1.27, 3.59)	< 0.001

^a^Adjusted for gender, age group, nationality, education, occupation, marital status, average monthly household income, current and past smoking status, and elevated blood pressure (WHO)

^b^Adjusted for gender, age group, nationality, education, occupation, marital status, average monthly household income, current and past smoking status, moderate-intensity physical activity and elevated blood pressure (WHO)

### Relative odds of overweight, obesity and central obesity by age group and gender

**Fig. 4:**
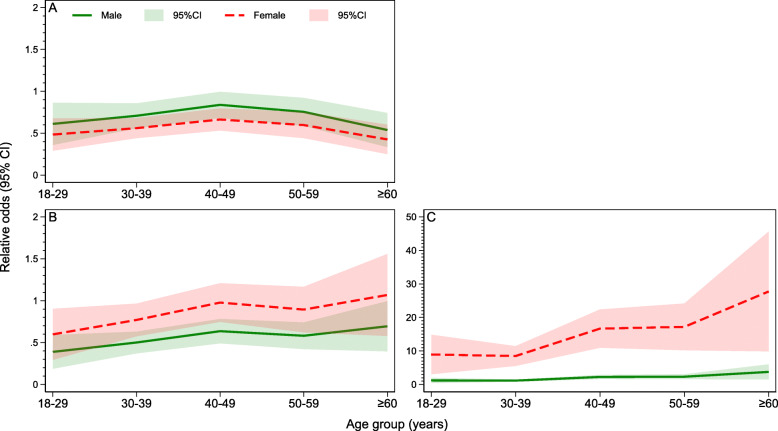
Relative odds of (**a**) overweight; **b** obesity and (**c**) central obesity (WC) across age groups by gender from fitted multivariable regression

Figure 4 shows the relative odds of overweight, obesity and central obesity. The gender difference in the trend of overweight and obesity across age groups was retained even after adjustment for socio-demographic factors. The relative odds of overweight was higher for men than for women, and for obesity higher for women than for men. For central obesity, there was a steep rise with increasing age in the relative odds of central obesity in women. In men, increases in central obesity across increasing age groups was little after accounting for other socio-demographic factors.

## Discussion

This representative study of predominant Kuwaiti and non-Kuwaiti population groups indicates a high prevalence of overweight, obesity and central obesity in Kuwait. Some 83% of participants were overweight or obese, and 74% had central obesity. This prevalence reveals a large national burden of overweight/obesity and central obesity in Kuwait. Men were more overweight than women, while women were more obese than men. Non-Kuwaiti were more overweight than Kuwaiti, while Kuwaiti were more obese than non-Kuwaiti. This finding is consistent with a recent report specifically for Kuwait [9] as well as earlier reports in other countries in the GCC, including Qatar [23], Saudi Arabia [24, 25], United Arab Emirates [26] and Bahrain [27]. Earlier cultural practices whereby women were discouraged from engaging in voluntary exercise, and valued for plumpness may, in combination with increasing consumption of calorie dense western foods, be a contributing factor to higher prevalence of obesity in women compared to men [28–30].

The trend to greater levels of overweight and obesity with advancing age has been studied extensively [31–33] and is evident also in our results. It is known that the population dynamics in many high-income countries have seen many of these countries witnessing high increase in life expectancy. However, the increase in life expectancy is often accompanied with additional years of susceptibility to various chronic conditions associated with overweight and obesity in later life [34]. This trend heralds a major burden posed to the healthcare system in managing a large elderly population with multiple comorbidities. Therefore, appropriate public health preventive measures to reduce overweight, obesity and or comorbidities in later life will aid in reducing healthcare costs. This is of relevance given that about 38 and 27% of study population within the age group of 18–29 years are already overweight and obese, respectively, with 50% having central obesity, and the likelihood that these rates will rise with age.

Socio-economic status is well known to influence risk factors for cardiovascular disease including overweight and obesity. The extent to which overweight/obesity associate with socio-economic indices depends on the economic development of the country [35–39]. Evidence in developed western countries suggests an inverse relationship between socio-economic indices and overweight/obesity among the adult population [40–43]. However, in developing countries, and in other high-income countries experiencing an epidemiological transition of obesity, the relationship has been inconsistent for many years [44]. An earlier report from China found that lower educational level and higher income were risk factors for obesity [45], while another found no evidence in the relationship [38]. Our study found that individuals within the higher income group were less likely to be overweight. This could be explained by the notion that as countries develop economically with rising prevalence of overweight, a larger proportion of overweight populations becomes relatively poor [46]. This association was the opposite for obesity, whereby the higher income group were more likely to be obese than the lower income group. This is because 78% (weighted) of participants were employed with a significant proportion earning above 500 Kuwaiti Dinah monthly per household (Table S1) – many of whom are expatriates or locals in high income employments. Several possible drivers of this patterning between overweight and obesity is attributed to a strong culture of physical inactivity and smoking [47], and beliefs that view individuals with obese physique as appealing and sign of affluence [48]. Also, the unemployed were less likely to be overweight and obese compared to those employed, after accounting for other factors.

Twenty-two percent and 18% of the sampled population were current and past smokers, respectively, and for each smoking history, men were more represented than women. An earlier study in Kuwait and Bahrain reported similar prevalence rates for smoking and differences across gender, although considerably higher for Kuwait than Bahrain [49]. Gender-specific prevalence rates in our study are lower, however, than those reported by RR Hamadeh [50] for the GCC countries in the 90s. Our results from adjusted analyses found that a history of smoking was directly associated with higher odds of obesity defined by BMI and central obesity. For current smokers, a stronger association with smoking was found for central obesity than for obesity defined per BMI. Our findings are consistent with a study from UK general population of 499,504 adults, which found that smoking was strongly associated with higher risk of obesity [51].

Estimates of physical activity were low. Weighted moderate and vigorous physical inactivity levels were 91 and 98%. This suggests that nearly all participants were physically inactive, in concordance with the report of the WHO that indicates that Kuwait, in conjunction with Iraq and Saudi Arabia were among the countries in the world with the highest prevalence of physical inactivity [52]. Our study further found that participants reporting physical activity, had a lower rate of obesity. Programmes to improve physical activity in Kuwait will arguably be integral to reducing obesity. It is important that governments integrate physical activity in the NCD prevention programmes at primary health care services.

Relationship between educational attainment and obesity are known to depend on the economic development of a country. Our findings show an inverse relationship between educational attainment and obesity and central obesity, aligning with the findings of a systematic review on educational attainment and obesity, where an inverse relationship was noted for all studies conducted in higher-income countries like Kuwait [53]. Therefore, educational policies to improve physical activity, healthful diet and reduction in smoking habits should be integral in efforts to reduce the prevalence of overweight/obesity in Kuwait.

### Strengths and limitations of the study

Large sample size and nationally representative study population are important strengths of our study. Our evaluation of different anthropometric indices enabled assessment of different types of obesity defined per established criteria. Our comparison of Kuwaiti and non-Kuwaiti resident in Kuwait is unique and has not been previously assessed. A limitation is that some factors were collected via questionnaire, and both recall, and desirability biases are possible.

## Conclusion

This study documents a high prevalence of overweight and obesity in the Kuwaiti population. A high prevalence of overweight/obesity among the young age group 18–29 years and unusually high prevalence of physical inactivity underlines the need for early intervention and a life course perspective to reduce and prevent obesity and overweight. Given that education was associated with reduced risk of obesity, integrating mandatory physical education to the school curriculum, and promoting the creation of public parks, recreation spaces to promote physical activities may play a vital role in the early prevention of overweight/obesity in Kuwait. These findings are crucial for public health advocates and policymakers, especially given the high burden of overweight and obesity in the Kuwait population, as they can guide the development of strategies, or action plans to reduce overweight and obesity, and physical inactivity in the population.

## Supplementary Information

**Additional file 1: Supplementary Tables. Table S1**. Study characteristics stratified by age group. **Table S2**. Study characteristics stratified by nationality.

## Data Availability

The raw dataset analysed in this study is not publicly available. It can be provided on reasonable request to Dasman Diabetes Institute through the corresponding author and in line with the provisions of the ethics committee.

## Abbreviations

WHO: World Health Organisation
STEPwise: Approach to Surveillance of noncommunicable diseases (STEPS)
BMI: Body mass index
WC: Waist circumference
OW: Overweight
OB: Obesity
GCC: Gulf Cooperation Council
GHO: Global Health Observatory
IDF: International Diabetes Federation
PACI: Public Authority of Civil Information
ACC/AHA: American College of Cardiology/American Heart Association Hypertension
CI: Confidence interval
KD: Kuwait Dinah
AOR: Adjusted odds ratio
