# Supplementary material for: Prevalence of overweight and obesity, and associations with socio-demographic factors in Kuwait
Source: BMC Public Health. 2021 Apr 7;21:667. doi: 10.1186/s12889-021-10692-1 (PMC8028185; doi:10.1186/s12889-021-10692-1)
Supplement: Supplementary file 1 — Additional file 1: Supplementary Tables. Table S1. Study characteristics stratified by age group. Table S2. Study characteristics stratified by nationality. [file 12889_2021_10692_MOESM1_ESM.docx]

**Supplementary Tables**

**Table S1. Study characteristics stratified by age group.**

|  | **Age group (years) Unweighted** | | | | | **Age group (years) Weighted** | | | | |
| --- | --- | --- | --- | --- | --- | --- | --- | --- | --- | --- |
| **Characteristics** | **18-29** | **30-39** | **40-49** | **50-59** | **≥60** | **18-29** | **30-39** | **40-49** | **50-59** | **≥60** |
|  | **N=372** | **N=1,306** | **N=1,808** | **N=1,027** | **N=388** | **N=254440** | **N=818084** | **N=1032876** | **N=527854** | **N=163020** |
|  | n (%) | n (%) | n (%) | n (%) | n (%) | n (%) | n (%) | n (%) | n (%) | n (%) |
| Age | 26.2 (2.6) | 35.0 (2.8) | 44.3 (2.9) | 53.7 (2.8) | 64.6 (4.6) | 26.1 (0.2) | 34.8 (0.1) | 44.2 (0.1) | 53.6 (0.1) | 64.3 (0.3) |
| **Nationality** |  |  |  |  |  |  |  |  |  |  |
| Kuwaiti | 80 (21.5) | 352 (27.0) | 625 (34.6) | 431 (42.0) | 201 (51.8) | 43108 (16.9) | 153683 (18.8) | 254207 (24.6) | 154286 (29.2) | 65147 (40.0) |
| non-Kuwaiti, specify | 292 (78.5) | 954 (73.0) | 1,183 (65.4) | 596 (58.0) | 187 (48.2) | 211332 (83.1) | 664401 (81.2) | 778669 (75.4) | 373568 (70.8) | 97872 (60.0) |
| **Ethnicity** |  |  |  |  |  |  |  |  |  |  |
| Arab | 233 (63.0) | 779 (59.8) | 1,191 (66.0) | 711 (69.6) | 319 (82.6) | 159008 (62.8) | 448037 (54.9) | 570883 (55.3) | 326476 (62.2) | 117032 (72.4) |
| Iranian | 8 (2.2) | 40 (3.1) | 81 (4.5) | 32 (3.1) | 7 (1.8) | 3936 (1.6) | 27771 (3.4) | 69894 (6.8) | 13045 (2.5) | 3338 (2.1) |
| South Asian | 84 (22.7) | 339 (26.0) | 370 (20.5) | 208 (20.4) | 46 (11.9) | 65662 (25.9) | 242204 (29.7) | 262579 (25.4) | 128791 (24.6) | 25376 (15.7) |
| Southeast Asia | 43 (11.6) | 141 (10.8) | 147 (8.1) | 65 (6.4) | 11 (2.8) | 24217 (9.6) | 97099 (11.9) | 119816 (11.6) | 52202 (10.0) | 8248 (5.1) |
| Other | 2 (0.5) | 4 (0.3) | 15 (0.8) | 6 (0.6) | 3 (0.8) | 519 (0.2) | 1616 (0.2) | 8667 (0.8) | 4080 (0.8) | 7584 (4.7) |
| **Average Income** |  |  |  |  |  |  |  |  |  |  |
| ≤500 | 157 (53.8) | 421 (39.9) | 497 (33.0) | 223 (26.8) | 45 (15.7) | 108986 (55.2) | 317049 (49.4) | 375135 (44.1) | 146585 (35.9) | 23690 (19.3) |
| >500-1500 | 79 (27.1) | 368 (34.8) | 511 (33.9) | 249 (30.0) | 109 (38.0) | 57571 (29.2) | 202547 (31.5) | 274348 (32.2) | 119303 (29.2) | 55806 (45.4) |
| >1500 | 56 (19.2) | 267 (25.3) | 499 (33.1) | 359 (43.2) | 133 (46.3) | 30824 (15.6) | 122451 (19.1) | 201756 (23.7) | 142566 (34.9) | 43373 (35.3) |
| **Education** |  |  |  |  |  |  |  |  |  |  |
| Illiterate | 1 (0.3) | 6 (0.5) | 10 (0.6) | 17 (1.7) | 23 (5.9) | 838 (0.3) | 8927 (1.1) | 8288 (0.8) | 23559 (4.5) | 7367 (4.5) |
| Read and write | 36 (9.7) | 180 (13.8) | 286 (15.9) | 216 (21.1) | 111 (28.7) | 38255 (15.0) | 133546 (16.3) | 195650 (19.0) | 117086 (22.3) | 44644 (27.5) |
| Secondary School | 92 (24.7) | 308 (23.6) | 466 (25.8) | 253 (24.7) | 79 (20.4) | 61534 (24.2) | 213832 (26.2) | 262134 (25.4) | 127793 (24.4) | 33703 (20.8) |
| University | 220 (59.1) | 705 (54.1) | 921 (51.1) | 466 (45.6) | 150 (38.8) | 134984 (53.1) | 406601 (49.8) | 499802 (48.4) | 221776 (42.3) | 67035 (41.3) |
| High studies | 23 (6.2) | 105 (8.1) | 121 (6.7) | 71 (6.9) | 24 (6.2) | 18829 (7.4) | 54081 (6.6) | 65964 (6.4) | 34522 (6.6) | 9549 (5.9) |
| **Occupation** |  |  |  |  |  |  |  |  |  |  |
| Employed | 305 (82.2) | 1,096 (84.2) | 1,454 (80.8) | 641 (62.9) | 139 (36.2) | 199220 (78.5) | 679145 (83.8) | 857174 (83.2) | 357580 (68.3) | 81443 (50.6) |
| Student, not employed | 25 (6.7) | 10 (0.8) | 4 (0.2) | 1 (0.1) | 0 (0.0) | 19468 (7.7) | 3853 (0.5) | 1051 (0.1) | 260 (0.0) | 0 |
| Housewife, not employed | 30 (8.1) | 180 (13.8) | 225 (12.5) | 107 (10.5) | 77 (20.1) | 17262 (6.8) | 119827 (14.8) | 126846 (12.3) | 69534 (13.3) | 25605 (15.9) |
| Retired | 1 (0.3) | 5 (0.4) | 95 (5.3) | 247 (24.2) | 145 (37.8) | 317 (0.1) | 2539 (0.3) | 37992 (3.7) | 87168 (16.7) | 46947 (29.1) |
| Unemployed | 10 (2.7) | 11 (0.8) | 22 (1.2) | 23 (2.3) | 23 (6.0) | 17559 (6.9) | 4890 (0.6) | 7736 (0.8) | 8696 (1.7) | 7063 (4.4) |
| **Marital status** |  |  |  |  |  |  |  |  |  |  |
| Never married | 193 (51.9) | 115 (8.8) | 48 (2.7) | 25 (2.4) | 7 (1.8) | 129203 (50.8) | 82678 (10.1) | 32221 (3.1) | 11855 (2.2) | 2066 (1.3) |
| currently married | 171 (46.0) | 1,147 (87.8) | 1,684 (93.2) | 954 (92.9) | 329 (84.8) | 121744 (47.8) | 717957 (87.8) | 966950 (93.6) | 495213 (93.8) | 142814 (87.6) |
| Divorced | 8 (2.2) | 41 (3.1) | 53 (2.9) | 28 (2.7) | 9 (2.3) | 3493 (1.4) | 16555 (2.0) | 23760 (2.3) | 13182 (2.5) | 2873 (1.8) |
| Widowed | 0 (0.0) | 3 (0.2) | 22 (1.2) | 20 (1.9) | 43 (11.1) | 0 (0.0) | 894 (0.1) | 9685 (0.9) | 7603 (1.4) | 15266 (9.4) |
| **Current smoker** |  |  |  |  |  |  |  |  |  |  |
| No | 266 (71.5) | 977 (74.9) | 1,354 (75.1) | 802 (78.4) | 330 (85.3) | 184480 (72.5) | 638061 (78.1) | 789227 (76.5) | 426985 (81.3) | 136796 (84.3) |
| Yes | 106 (28.5) | 327 (25.1) | 449 (24.9) | 221 (21.6) | 57 (14.7) | 69960 (27.5) | 178926 (21.9) | 242351 (23.5) | 98330 (18.7) | 25503 (15.7) |
| **Past smoker** |  |  |  |  |  |  |  |  |  |  |
| No | 238 (89.8) | 875 (89.1) | 1,162 (85.5) | 643 (79.7) | 245 (73.6) | 166994 (90.4) | 570509 (88.9) | 624653 (79.3) | 331079 (77.4) | 101199 (73.5) |
| Yes | 27 (10.2) | 107 (10.9) | 197 (14.5) | 164 (20.3) | 88 (26.4) | 17804 (9.6) | 71184 (11.1) | 162763 (20.7) | 96703 (22.6) | 36561 (26.5) |
| **Vigorous-intensity physical activity** |  |  |  |  |  |  |  |  |  |  |
| No | 365 (98.1) | 1,278 (98.0) | 1,766 (97.9) | 1,002 (97.9) | 384 (99.2) | 250888 (98.6) | 795428 (97.4) | 1003400 (97.2) | 513473 (97.7) | 159575 (98.3) |
| Yes | 7 (1.9) | 26 (2.0) | 38 (2.1) | 21 (2.1) | 3 (0.8) | 3551 (1.4) | 21559 (2.6) | 28438 (2.8) | 11842 (2.3) | 2723 (1.7) |
| **Moderate-intensity physical activity** |  |  |  |  |  |  |  |  |  |  |
| No | 320 (86.0) | 1,178 (90.4) | 1,677 (93.2) | 952 (93.1) | 362 (93.5) | 221323 (87.0) | 722573 (88.5) | 944484 (91.8) | 490106 (93.3) | 144590 (89.1) |
| Yes | 52 (14.0) | 125 (9.6) | 123 (6.8) | 71 (6.9) | 25 (6.5) | 33117 (13.0) | 93576 (11.5) | 84581 (8.2) | 35208 (6.7) | 17709 (10.9) |
| **Elevated blood pressure (ACC/AHA)** |  |  |  |  |  |  |  |  |  |  |
| No | 310 (83.6) | 946 (72.5) | 1,199 (66.5) | 558 (54.6) | 220 (56.8) | 212099 (83.4) | 548927 (67.2) | 658603 (63.8) | 262000 (49.9) | 85163 (52.5) |
| Yes | 61 (16.4) | 358 (27.5) | 604 (33.5) | 464 (45.4) | 167 (43.2) | 42081 (16.6) | 268060 (32.8) | 372976 (36.2) | 263055 (50.1) | 77135 (47.5) |
| **Elevated blood pressure (WHO)** |  |  |  |  |  |  |  |  |  |  |
| No | 352 (94.9) | 1,202 (92.2) | 1,583 (87.8) | 839 (82.1) | 332 (85.8) | 239442 (94.2) | 746484 (91.4) | 887244 (86.0) | 412730 (78.6) | 133986 (82.6) |
| Yes | 19 (5.1) | 102 (7.8) | 220 (12.2) | 183 (17.9) | 55 (14.2) | 14738 (5.8) | 70503 (8.6) | 144335 (14.0) | 112325 (21.4) | 28313 (17.4) |
| **Height (m);** mean (SD) | 1.7 (0.1) | 1.7 (0.1) | 1.7 (0.1) | 1.6 (0.1) | 1.6 (0.1) | 1.7 (0.0) | 1.7 (0.0) | 1.7 (0.0) | 1.6 (0.0) | 1.6 (0.0) |
| **Weight (kg);** mean (SD) | 76.6 (22.0) | 80.5 (17.9) | 83.7 (18.0) | 84.0 (16.6) | 82.9 (15.9) | 79.0 (1.7) | 80.5 (0.8) | 82.9 (0.6) | 83.6 (1.1) | 81.8 (0.9) |
| **Waist Circumference (cm);** mean (SD) | 89.5 (14.9) | 94.5 (12.5) | 98.4 (13.1) | 100.8 (12.6) | 104.6 (13.0) | 91.5 (1.4) | 95.0 (0.5) | 98.5 (0.5) | 101.0 (0.7) | 103.6 (0.8) |
| **Hip Circumference (cm);** mean (SD) | 102.9 (12.8) | 105.7 (11.1) | 107.3 (11.8) | 107.8 (11.7) | 109.8 (11.7) | 103.6 (1.0) | 105.3 (0.5) | 106.1 (0.4) | 107.0 (0.6) | 108.2 (0.7) |
| **Waist to height ratio;** mean (SD) | 0.5 (0.1) | 0.6 (0.1) | 0.6 (0.1) | 0.6 (0.1) | 0.6 (0.1) | 0.5 (0.0) | 0.6 (0.0) | 0.6 (0.0) | 0.6 (0.0) | 0.6 (0.0) |

**Table S2. Study characteristics stratified by nationality.**

| Characteristics | Unweighted | | Weighted | |
| --- | --- | --- | --- | --- |
|  | Kuwaiti | non-Kuwaiti | Kuwaiti | non-Kuwaiti |
|  | N=1,689 | N=3,212 | N=670432 | N=2125842 |
|  | n (%) | n (%) | n (%) | n (%) |
| **Average Income** |  |  |  |  |
| ≤500 | 4 (0.3) | 1,339 (52.8) | 1476 (0.3) | 969970 (58.8) |
| >500-1500 | 309 (21.5) | 1,007 (39.7) | 131862 (23.1) | 577712 (35.0) |
| >1500 | 1,122 (78.2) | 192 (7.6) | 438569 (76.7) | 102401 (6.2) |
| **Education** |  |  |  |  |
| Illiterate | 25 (1.5) | 32 (1.0) | 9538 (1.4) | 39442 (1.9) |
| Read and write | 242 (14.3) | 587 (18.3) | 98403 (14.7) | 430778 (20.3) |
| Secondary School | 251 (14.9) | 947 (29.6) | 99687 (14.9) | 599309 (28.3) |
| University | 1,058 (62.6) | 1,404 (43.9) | 421229 (62.8) | 908970 (42.9) |
| High studies | 113 (6.7) | 231 (7.2) | 41576 (6.2) | 141369 (6.7) |
| **Occupation** |  |  |  |  |
| Employed | 1,042 (61.7) | 2,593 (81.3) | 440297 (65.7) | 1734264 (82.2) |
| Student, not employed | 19 (1.1) | 21 (0.7) | 7255 (1.1) | 17377 (0.8) |
| Housewife, not employed | 99 (5.9) | 520 (16.3) | 35694 (5.3) | 323380 (15.3) |
| Retired | 474 (28.1) | 19 (0.6) | 168185 (25.1) | 6778 (0.3) |
| Unemployed | 54 (3.2) | 35 (1.1) | 18387 (2.7) | 27558 (1.3) |
| **Marital status** |  |  |  |  |
| Never married | 55 (3.3) | 333 (10.4) | 24263 (3.6) | 233761 (11.0) |
| currently married | 1,512 (89.5) | 2,773 (86.4) | 600405 (89.6) | 1844274 (86.8) |
| Divorced | 75 (4.4) | 64 (2.0) | 30196 (4.5) | 29668 (1.4) |
| Widowed | 47 (2.8) | 41 (1.3) | 15568 (2.3) | 17880 (0.8) |
| **Current smoker** |  |  |  |  |
| No | 1,351 (80.0) | 2,378 (74.3) | 534533 (79.7) | 1641016 (77.4) |
| Yes | 338 (20.0) | 822 (25.7) | 135899 (20.3) | 479171 (22.6) |
| **Past smoker** |  |  |  |  |
| No | 1,201 (87.0) | 1,962 (83.0) | 476055 (87.3) | 1318377 (80.7) |
| Yes | 180 (13.0) | 403 (17.0) | 69337 (12.7) | 315678 (19.3) |
| **Vigorous-intensity physical activity** |  |  |  |  |
| No | 1,684 (99.7) | 3,111 (97.2) | 668163 (99.7) | 2054601 (96.9) |
| Yes | 5 (0.3) | 90 (2.8) | 2269 (0.3) | 65845 (3.1) |
| **Moderate-intensity physical activity** |  |  |  |  |
| No | 1,683 (99.6) | 2,806 (87.8) | 668178 (99.7) | 1854898 (87.6) |
| Yes | 6 (0.4) | 390 (12.2) | 2254 (0.3) | 261937 (12.4) |
| **Elevated blood pressure (ACC/AHA)** |  |  |  |  |
| No | 1,267 (75.0) | 1,966 (61.5) | 513900 (76.7) | 1252892 (59.1) |
| Yes | 422 (25.0) | 1,232 (38.5) | 156532 (23.3) | 866775 (40.9) |
| **Elevated blood pressure (WHO)** |  |  |  |  |
| No | 1,582 (93.7) | 2,726 (85.2) | 632539 (94.3) | 1787347 (84.3) |
| Yes | 107 (6.3) | 472 (14.8) | 37893 (5.7) | 332320 (15.7) |
| **Height (m);** mean (SD) | 1.7 (0.1) | 1.7 (0.1) | 45.4 (0.3) | 41.9 (0.3) |
| **Weight (kg);** mean (SD) | 85.1 (17.4) | 80.8 (18.1) | 1.7 (0.0) | 1.7 (0.0) |
| **Waist Circumference (cm);** mean (SD) | 100.2 (12.9) | 96.3 (13.6) | 84.8 (0.5) | 81.0 (0.5) |
| **Hip Circumference (cm);** mean (SD) | 109.5 (11.9) | 105.5 (11.5) | 99.7 (0.3) | 97.0 (0.4) |
| **Waist to height ratio;** mean (SD) | 0.6 (0.1) | 0.6 (0.1) | 109.2 (0.3) | 104.9 (0.3) |
